# O Preço Oculto do Acesso Negado: Insuficiência Cardíaca Avançada na América Latina

**DOI:** 10.36660/abc.20250609

**Published:** 2025-11-27

**Authors:** Fernanda Almeida Andrade

**Affiliations:** 1 Instituto do Coração do Hospital das Clínicas da Faculdade de Medicina da Universidade de São Paulo São Paulo SP Brasil Instituto do Coração do Hospital das Clínicas da Faculdade de Medicina da Universidade de São Paulo, São Paulo, SP – Brasil; 2 Escola Paulista de Medicina da Universidade Federal de São Paulo São Paulo SP Brasil Escola Paulista de Medicina da Universidade Federal de São Paulo (EPM/UNIFESP), São Paulo, SP – Brasil

**Keywords:** Recursos Financeiros em Saúde, Custos e Análise de Custo, Insuficiência Cardíaca, América Latina

A insuficiência cardíaca avançada (IC avançada) se configura como a expressão terminal da síndrome, marcada pela refratariedade às estratégias farmacológicas e não farmacológicas atualmente disponíveis. Trata-se de uma condição que concentra elevadas taxas de morbimortalidade e um consumo desproporcional de recursos assistenciais, impondo ônus significativo tanto aos sistemas de saúde quanto às famílias. Em face do envelhecimento populacional e do aumento da sobrevida de indivíduos com cardiopatias crônicas, projeta-se uma expansão progressiva da prevalência dessa condição, com impacto crescente sobre custos diretos (hospitalizações, terapias avançadas, suporte intensivo) e indiretos (perda de produtividade e dependência social).^1,2^

Nos países de alta renda, intervenções de alto custo como o transplante cardíaco e a assistência circulatória mecânica de longa duração se consolidaram como pilares terapêuticos capazes de alterar a história natural da doença.^3^ O transplante se mantém como padrão-ouro de tratamento, embora sua aplicação prática esteja limitada pela escassez de doadores e pela elevada proporção de pacientes inelegíveis devido às contraindicações clínicas. Nesse vácuo terapêutico, os dispositivos de assistência ventricular esquerda (DAVEs) emergiram como alternativas progressivamente mais eficazes, sobretudo na modalidade de terapia de destino. A introdução de tecnologias de fluxo contínuo e, mais recentemente, de bombas magneticamente levitadas do HeartMate 3, redefiniu o prognóstico desses pacientes, oferecendo sobrevida superior a cinco anos em centros especializados.^4^

O panorama latino-americano se distancia de forma marcante das experiências norte-americana e europeia. Nos Estados Unidos, o número de implantes de DAVEs alcançou, já em 2019, cifras comparáveis às do transplante cardíaco, enquanto na Europa a adoção desses dispositivos segue trajetória ascendente e contínua.^5,6^ Em contrapartida, em grande parte dos países da América Latina, sua utilização permanece incipiente, restrita a protocolos de pesquisa ou acessível apenas a segmentos da saúde suplementar. Esse contraste decorre de barreiras estruturais e econômicas persistentes, como o subfinanciamento histórico dos sistemas públicos, a acentuada desigualdade de acesso entre regiões e a inexistência de programas nacionais voltados ao suporte circulatório mecânico. O resultado é um hiato assistencial que acentua as disparidades já existentes no manejo da insuficiência cardíaca avançada.^7^

Goldraich et al. (2025) trouxeram evidência inédita ao estimar, em um hospital público brasileiro, o custo real do manejo clínico de pacientes com IC avançada candidatos a DAVEs, mas sem acesso à tecnologia. Utilizando a metodologia de *time-driven activity-based costing* - na qual, com base no ciclo de cuidado do paciente, os recursos consumidos são medidos de acordo com o tempo demandado por cada paciente (custo unitário do cuidado e tempo necessário para realização de uma atividade), os autores acompanharam 20 pacientes consecutivos (idade média 51 anos, predominantemente homens), desde a indicação para DAVEs até o óbito ou fim do seguimento. A sobrevida global foi de apenas 40% em 15 meses de acompanhamento, com um custo médio por paciente de Int$ 120.457 (Int$ = dólar internacional, paridade de poder de compra), sendo 72% relacionado a hospitalizações. Observou-se ainda que os pacientes em perfis INTERMACS mais graves demandaram quase 70% mais recursos que aqueles sem necessidade de inotrópicos (Int$ 156.457 vs. Int$ 91.003). Esses achados expõem de forma objetiva o paradoxo latino-americano: altos custos acumulados com hospitalizações repetidas e baixa efetividade clínica, em contraste com a possibilidade de destinar esses mesmos recursos a terapias avançadas que comprovadamente modificam o prognóstico. O estudo fornece subsídio crítico para análises de custo-efetividade e reforça a urgência de discutir a incorporação racional de dispositivos em sistemas públicos de saúde na região.^8^

Importa destacar que a ausência de terapias de alta complexidade não elimina custos, apenas os redistribui. Pacientes sem acesso ao transplante ou a dispositivos de assistência circulatória permanecem submetidos a hospitalizações repetidas, ao uso prolongado de agentes inotrópicos e a intervenções de resgate com baixo impacto prognóstico. Esse padrão de cuidado fragmentado gera dispêndios significativos para os sistemas de saúde, sem proporcionar ganhos proporcionais em sobrevida ou qualidade de vida. Tanto as Diretrizes Brasileiras de Insuficiência Cardíaca quanto as recomendações internacionais da ESC enfatizam a importância da identificação precoce de candidatos a terapias avançadas e da criação de fluxos assistenciais organizados; no entanto, tais recomendações seguem pouco implementadas em países de renda média, perpetuando a ineficiência do modelo vigente.^9,10^

Russo et al. (2008), ao analisar o braço clínico do ensaio REMATCH, evidenciaram que os custos da IC avançada se concentram de forma dramática no fim da vida: o gasto médio por paciente alcançou USD 156.169 nos últimos dois anos, sendo mais da metade (50,5%; USD 78.880) restrita aos seis meses finais. Essa magnitude contrasta com o perfil de custos da insuficiência cardíaca estável. Em Silva Etges et al. (2023), os dispêndios anuais em pacientes ambulatoriais variaram de apenas Int$ 510 (NYHA I) a Int$ 1.334 (NYHA III/IV), com predominância de exames e procedimentos, que responderam por até 74% do total. A discrepância entre estágios distintos da doença ilustra não apenas a intensidade do consumo de recursos nos casos mais graves, mas também a oportunidade perdida de intervenção precoce. Esses achados reforçam o argumento de que a avaliação de custo-efetividade das terapias avançadas não deve ser negligenciada: compreender o peso financeiro real da progressão da IC pode orientar políticas públicas mais racionais e favorecer a incorporação de tecnologias em contextos de maior impacto social.^11,12^

Nesse contexto, se impõe a necessidade de quantificar, de forma metodologicamente rigorosa, o custo real do manejo clínico isolado da insuficiência cardíaca avançada em sistemas com recursos limitados. Somente com dados robustos será possível dimensionar adequadamente o peso econômico da doença e desenvolver análises de custo-efetividade que orientem políticas públicas, favoreçam decisões mais racionais sobre incorporação tecnológica e reduzam desigualdades de acesso.^13^

Negar o acesso a tecnologias capazes de modificar o curso da insuficiência cardíaca avançada suscita não apenas questões econômicas, mas também dilemas éticos profundos. Em sistemas públicos universais, a decisão de excluir terapias comprovadas por seu alto custo precisa ser debatida à luz do princípio da equidade: até que ponto é aceitável condenar pacientes à elevada mortalidade e baixa qualidade de vida quando existem alternativas, ainda que caras, com potencial de prolongar a sobrevida e reduzir hospitalizações repetidas? Esse desafio não deve ser enfrentado isoladamente. A criação de consórcios latino-americanos de insuficiência cardíaca poderia viabilizar registros regionais robustos, padronizar critérios de elegibilidade e, sobretudo, fortalecer o poder de negociação coletiva com fabricantes para redução de preços. Cabe propor um caminho pragmático e incremental: lançar programas-piloto em centros de referência, fomentar estudos multicêntricos de custo-efetividade adaptados à realidade da região e estabelecer metas graduais de incorporação de dispositivos de assistência ventricular esquerda nos países de renda média. Só assim será possível transformar o paradoxo atual - custos altos com baixa efetividade, em uma agenda de saúde pública mais justa, sustentável e humanizada.

## References

[1] Subramaniam AV, Weston SA, Killian JM, Schulte PJ, Roger VL, Redfield MM (2022). Development of Advanced Heart Failure: A Population-Based Study. Circ Heart Fail.

[2] Mehra MR, Canter CE, Hannan MM, Semigran MJ, Uber PA, Baran DA (2016). The 2016 International Society for Heart Lung Transplantation Listing Criteria for Heart Transplantation: A 10-Year Update. J Heart Lung Transplant.

[3] Molina EJ, Shah P, Kiernan MS, Cornwell WK, Copeland H, Takeda K (2021). The Society of Thoracic Surgeons Intermacs 2020 Annual Report. Ann Thorac Surg.

[4] Colvin M, Smith JM, Ahn Y, Skeans MA, Messick E, Goff R (2021). OPTN/SRTR 2019 Annual Data Report: Heart. Am J Transplant.

[5] Nayak A, Hall SA, Uriel N, Goldstein DJ, Cleveland JC, Cowger JA (2023). Predictors of 5-Year Mortality in Patients Managed with a Magnetically Levitated Left Ventricular Assist Device. J Am Coll Cardiol.

[6] Mehra MR, Nayak A, Desai AS (2023). Life-Prolonging Benefits of LVAD Therapy in Advanced Heart Failure: A Clinician's Action and Communication Aid. JACC Heart Fail.

[7] Nayak A, Mehra MR (2022). Global Challenges in Left Ventricular Assist Device Therapy: A Tale Across Two Continents. Eur J Heart Fail.

[8] Goldraich LA, Etges APBS, Hastenteufel LCT, Lemos DM, Zuckermann A, Mehra MR (2025). The Cost of Medical Management in Advanced Heart Failure: A Latin American Perspective. Arq Bras Cardiol.

[9] Marcondes-Braga FG, Moura LAZ, Issa VS, Vieira JL, Rohde LE, Simões MV (2021). Emerging Topics Update of the Brazilian Heart Failure Guideline - 2021. Arq Bras Cardiol.

[10] McDonagh TA, Metra M, Adamo M, Gardner RS, Baumbach A, Böhm M (2021). 2021 ESC Guidelines for the Diagnosis and Treatment of Acute and Chronic Heart Failure. Eur Heart J.

[11] Russo MJ, Gelijns AC, Stevenson LW, Sampat B, Aaronson KD, Renlund DG (2008). The Cost of Medical Management in Advanced Heart Failure during the Final Two Years of Life. J Card Fail.

[12] Jorde UP, Saeed O, Koehl D, Morris AA, Wood KL, Meyer DM (2024). The Society of Thoracic Surgeons Intermacs 2023 Annual Report: Focus on Magnetically Levitated Devices. Ann Thorac Surg.

[13] Etges APBS, Liu HH, Jones P, Polanczyk CA (2023). Value-Based Reimbursement as a Mechanism to Achieve Social and Financial Impact in the Healthcare System. J Health Econ Outcomes Res.

