# Supplementary material for: O Preço Oculto do Acesso Negado: Insuficiência Cardíaca Avançada na América Latina
Source: Arq Bras Cardiol. 2025 Nov 27;122(10):e20250609. [Article in Portuguese] doi: 10.36660/abc.20250609 (PMC12671607; doi:10.36660/abc.20250609)
Supplement: Translation

# Translation: The Hidden Cost of Denied Access: Advanced Heart Failure in Latin America

**DOI:** 10.36660/abc.20250609i

**Authors:** Fernanda Almeida Andrade

**Affiliations:** 1 São Paulo SP Brazil Instituto do Coração do Hospital das Clínicas da Faculdade de Medicina da Universidade de São Paulo, São Paulo, SP – Brazil; 2 São Paulo SP Brazil Escola Paulista de Medicina da Universidade Federal de São Paulo (EPM/UNIFESP), São Paulo, SP – Brazil

**Keywords:** Financial Resources in Health, Costs and Cost Analysis, Heart Failure, Latin America

Advanced heart failure (AHF) represents the terminal stage of the syndrome, characterized by refractoriness to currently available pharmacological and non-pharmacological strategies. It is a condition associated with high morbidity and mortality rates and a disproportionate consumption of healthcare resources, imposing a substantial burden on both healthcare systems and families. With population aging and the increased survival of individuals with chronic heart disease, the prevalence of AHF is projected to grow steadily, with escalating impact on direct costs (hospitalizations, advanced therapies, intensive support) and indirect costs (loss of productivity and social dependency).^1,2^

In high-income countries, costly interventions such as heart transplantation and long-term mechanical circulatory support have become consolidated therapeutic pillars that can alter the natural history of the disease.^3^ Heart transplantation remains the gold standard of treatment, although its practical application is limited by donor shortages and a high proportion of clinically ineligible patients. Within this therapeutic gap, left ventricular assist devices (LVADs) have progressively emerged as increasingly effective alternatives, particularly as destination therapy. The introduction of continuous-flow technologies and, more recently, magnetically levitated pumps, such as the HeartMate 3, has redefined prognosis, providing survival beyond five years in specialized centers.^4^

The Latin American landscape differs markedly from North American and European experiences. In the United States, the number of LVAD implants had already reached figures comparable to those of heart transplantation in 2019, while in Europe, the adoption of these devices continues to follow a steady upward trajectory.^5,6^ In contrast, in most Latin American countries, their use remains incipient, often restricted to research protocols or limited to the private healthcare sector. This contrast reflects persistent structural and economic barriers, such as chronic underfunding of public systems, pronounced regional inequalities in access, and the absence of national programs dedicated to mechanical circulatory support. The result is a care gap that exacerbates preexisting disparities in the management of AHF.^7^

Goldraich LA et al. (2025) provided novel evidence by estimating, in a Brazilian public hospital, the real cost of clinical management of patients with advanced HF who were candidates for LVADs but lacked access to the technology. Using time-driven activity-based costing, in which the patient care cycle is mapped and resources consumed are measured according to the unit cost of care and the time required to perform each activity, the authors followed 20 consecutive patients (mean age 51 years, predominantly male) from the moment of LVAD indication until death or end of follow-up. Overall survival was only 40% after 15 months, with a mean cost per patient of Int$ 120,457 (Int$ = International Dollar, based on purchasing power parity), of which 72% was related to hospitalizations. Patients in more severe INTERMACS profiles demanded nearly 70% more resources compared with those not requiring inotropes (Int$ 156,457 vs. Int$ 91,003). These findings objectively expose the Latin American paradox: high cumulative costs driven by recurrent hospitalizations and low clinical effectiveness, in contrast with the potential to allocate similar resources to advanced therapies that have proven prognostic benefits. The study provides critical support for cost-effectiveness analyses and underscores the urgency of discussing the rational incorporation of such devices into public health systems in the region.^8^

It is important to emphasize that the absence of high-complexity therapies does not eliminate costs but merely redistributes them. Patients without access to transplantation or mechanical circulatory support remain subjected to repeated hospitalizations, prolonged use of inotropic agents, and rescue interventions with limited prognostic benefit. This fragmented pattern of care generates substantial expenditures for healthcare systems without yielding proportional gains in survival or quality of life. Both the Brazilian Heart Failure Guidelines and the ESC recommendations highlight the importance of early identification of candidates for advanced therapies and the establishment of organized referral pathways. However, such recommendations remain poorly implemented in middle-income countries, perpetuating inefficiencies in the current model.^9,10^

Russo et al. (2008), in the clinical arm of the REMATCH trial, demonstrated that the costs of advanced HF are dramatically concentrated at the end of life: the mean per-patient expenditure reached USD 156,169 in the last two years, with more than half (50.5%; USD 78,880) occurring in the final six months. This magnitude contrasts sharply with the cost profile of stable heart failure. In Silva Etges et al. (2023), annual expenditures for outpatients ranged from as little as Int$ 510 (NYHA I) to Int$ 1,334 (NYHA III/IV), with diagnostic tests and procedures accounting for up to 74% of the total. The discrepancy across disease stages illustrates not only the intensity of resource consumption among the most severe cases but also the missed opportunity for earlier intervention. These findings strengthen the argument that cost-effectiveness evaluation of advanced therapies should not be neglected: understanding the true financial burden of HF progression can guide more rational public policies and support the incorporation of technologies in contexts where they may have the greatest social impact.^11,12^

In this context, it becomes imperative to rigorously quantify the real cost of isolated clinical management of AHF in resource-limited systems. Only with robust data will it be possible to adequately assess the economic burden of the disease and to develop cost-effectiveness analyses that can inform public policy, support more rational decisions on technology adoption, and reduce inequities in access.^13^

Denying access to technologies capable of altering the course of AHF raises not only economic concerns but also profound ethical dilemmas. In universal public health systems, the decision to exclude proven therapies based on high cost must be debated through the lens of equity: to what extent is it acceptable to condemn patients to high mortality and poor quality of life when alternatives exist that, albeit expensive, have the potential to prolong survival and reduce recurrent hospitalizations? This challenge cannot be faced in isolation. The creation of Latin American heart failure consortia could enable robust regional registries, standardize eligibility criteria, and, most importantly, strengthen collective bargaining power with manufacturers to achieve price reductions. A pragmatic and incremental pathway should be pursued: implementing pilot programs in referral centers, fostering multicenter cost-effectiveness studies tailored to the region, and gradually establishing targets for the incorporation of LVADs in middle-income countries. Only in this way will it be possible to transform the current paradox of high costs with low effectiveness into a public health agenda that is fairer, more sustainable, and truly patient-centered.
